# Collapse and collision aware grasping for cluttered shelf picking

**DOI:** 10.3389/frobt.2026.1697561

**Published:** 2026-03-11

**Authors:** Abhinav Pathak, Kalaichelvi Venkatesan, Tarek Taha, Rajkumar Muthusamy

**Affiliations:** 1 Robotics Lab, Dubai Future Labs, Dubai, United Arab Emirates; 2 Birla Institute of Technology and Science, Dubai, United Arab Emirates

**Keywords:** collapse aware grasp planning, industrial automation, robotic manipulation, shelf picking, warehouse automation

## Abstract

In modern smart factories, automated shelf picking must deliver high throughput, flexibility, and safe human–robot collaboration. In these environments, efficient and safe retrieval of stacked objects is a significant challenge due to complex spatial dependencies and structural inter-dependencies. Traditional vision-based methods excel at object localization but often lack the physical reasoning required to predict the consequences of extraction, leading to unintended collisions and collapses. This paper proposes a collapse and collision-aware grasp planner that integrates dynamic physics simulations for robotic decision-making. Using a single image and depth map, an approximate 3D representation of the scene is reconstructed in a simulation environment, enabling the robot to evaluate different retrieval strategies before execution. Two approaches: 1) heuristic-based and 2) physics-based are proposed for both single-box extraction and shelf clearance tasks. Extensive real-world experiments on structured and unstructured box stacks, along with validation using datasets from existing databases, show that our physics-aware method significantly improves efficiency and success rates compared to baseline heuristics. A video demonstrating the real-world implementation of our proposed system is available at: https://youtu.be/GBWMiNIHUlU.

## 1 Introduction

**FIGURE 1:**
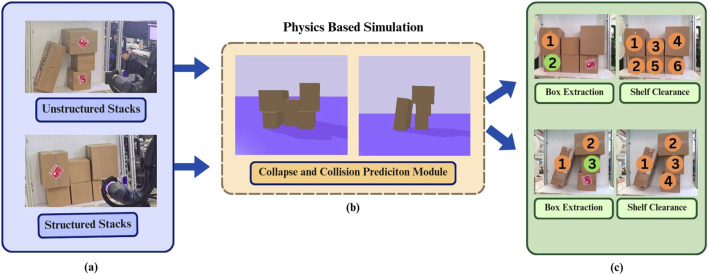
Cluttered shelf object retrieval **(a)** The autonomous robot observes the stacking complexities **(b)** using the perceived image and its features the robot grasp planner conducts physics driven simulation to predict collapses and collision for the retrieval tasks **(c)** grasp sequence for desired box extraction and clearing out all boxes from shelf.

The rapid growth of interest in Industry 4.0 and the integration of autonomous robotic systems into warehouse management has also skyrocketed; this has led to significant improvements in efficiency, particularly in tasks such as item retrieval, transportation, and inventory organization. A major challenge in such settings is the safe and efficient retrieval of target objects without destabilizing surrounding structures. Unlike traditional pick-and-place operations in robotics, stacked object retrieval involves complex spatial dependencies, as seen in Figure 1a, where the removal of one item can introduce unintended disturbances, leading to collapses or collisions that disrupt warehouse operations. Existing approaches to robotic object retrieval primarily treat the problem as a vision-based task, relying on object detection and segmentation to identify and extract items from storage.

Although these methods are excellent at identifying object locations, they often overlook the physical effects of removing an object. In particular, they do not simulate how forces are distributed to keep a stack stable, which can lead to unexpected structural collapses. This lack of predictive reasoning severely limits their applicability in real-world warehouse environments, where errors can cause operational delays, financial losses, and safety hazards.

In the context of stacked object retrieval, two primary failure modes must be addressed:

1. Collapses: A collapse is a cascading failure where the removal of one object disrupts the weight distribution of the stack, causing multiple objects to fall. This failure mode arises due to a loss of structural support, which can be difficult to predict without physics-aware reasoning. Unlike a direct collision, which involves immediate contact, a collapse can occur after an object is removed due to the gradual redistribution of forces within the stack.
2. Collisions: These occur when the robotic manipulator, end-effector, or target object makes unintended contact with adjacent objects during retrieval. Such interactions may cause minor position shifts or major toppling events, leading to downstream failures in structured storage. The problem is exacerbated by occlusions, where objects partially obstruct one another, reducing visibility and increasing the likelihood of contact.

**FIGURE 2:**
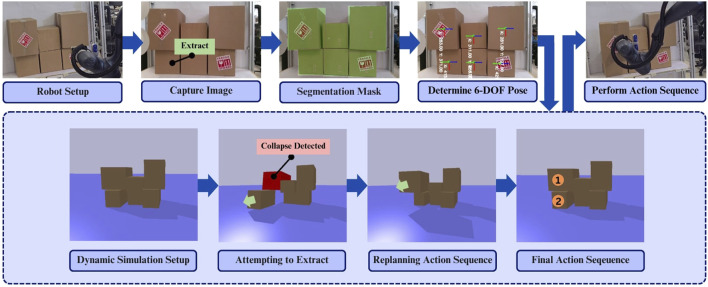
Overview of the proposed grasping pipeline using the physics-aware approach for safe cardboard box extraction.

This paper proposes a physics-based approach to model object interactions, enhancing retrieval accuracy and safety. Rather than relying solely on vision-based methods, this system aims to predict the consequences of retrieval actions using a physics-aware real-to-sim approach as in Figures 1b,c. With a single RGB and depth image, it can reconstruct an approximate 3D representation of the scene, allowing a robotic arm to evaluate different retrieval strategies before execution. By incorporating segmentation, bounding box estimation, centroid estimation, and depth sensing, it generates a virtual simulation where potential collapses and object movements can be predicted. This allows the robot to develop a form of ‘intuition’, understanding how the removal of an item will impact the surrounding objects as seen in Figure 2.

Our contributions can be summarized as follows:

1. A single-shot, collapse-collision aware grasp planner for cluttered shelf picking. The planner conducts physics simulations using the features extracted from the modeling image.
2. Two retrieval approaches, heuristic-based and physics-based, to perform single object extraction and shelf object clearance.
3. Extensive real-world experiments to validate and evaluate the planner and approaches under varying stacking complexities (structured and unstructured box stacks).
4. Scalability demonstration of the proposed methods on diverse datasets from large-scale databases.

The paper is structured as follows: Section 2 reviews related work in object retrieval, simulation-based planning, and physics-informed robotics. Section 3 details the proposed method, including the perception pipeline, simulation techniques, and decision-making framework. Experimental results are presented in Section 4, comparing the performance of the proposed approach in both simulation and real-world scenarios. Finally, Section 5 provides conclusions, discusses limitations, and outlines potential future research directions.

## 2 Related work

Recent advances in robotic manipulation leverage vision, learning, and physics to improve object extraction from clutter. Vision-based methods, like those by Chen et al. (2023) and Mahler et al. (2017), use deep neural networks and synthetic data for grasp planning but often neglect structural interactions among objects. Bejjani et al. (2021) proposed search strategies for occlusion by predicting object locations, while their learning frameworks (Bejjani et al., 2019) optimize actions in simulation. However, these methods struggle with real-world dynamics. Additionally, Bohg et al. (2014) reviewed various grasp planners for known, familiar, and unknown objects. Our collision and collapse predicting method can leverage already existing grasp planners. In this paper, we also propose our own simplified grasp planner.

Physics-informed approaches incorporate physical reasoning to improve safety and stability. Motoda et al. (2021) introduced a bi-manual planner that uses physics simulation to predict collapse, a concept extended in their later paper (Motoda et al., 2023) by modeling support relations for safe, multi-step extractions. Meanwhile, real-to-sim frameworks like Zook et al. (2024) reconstruct 3D scenes from modeling data to generate simulation tasks, though they focus more on task synthesis than on ensuring stability.

Other works integrate physics into learning pipelines. Physics-informed neural networks (Raissi et al., 2019; Ni and Qureshi, 2023) embed physical laws into their models but typically require extensive training and lack real-time adaptability. In contrast, Marchionna et al. (2023) combines instance segmentation with force sensing for precise block extraction in Jenga, while Banerjee et al. (2024) reviews the potential of physics-aware vision models.

Recent studies have explored hybrid approaches that combine vision, learning, and physics, integrating deep neural networks with physical simulation to capture visual cues and structural dynamics. However, these frameworks struggle with real-world adaptability and high computational costs.

Geometric reasoning and support relation analysis provide complementary approaches to understanding structural dependencies in object piles. Mojtahedzadeh et al. (2015) proposed a framework for extracting support relations using geometric features and static equilibrium principles from classical mechanics, enabling decision-making strategies that optimize expected costs when removing objects from randomly configured piles. Their approach addresses both fully detected and partially detected scenarios, leveraging machine learning techniques to estimate support probabilities under incomplete perception. Kartmann et al. (2018) extracted physically plausible support relations directly from visual information without requiring prior knowledge of physical properties such as mass distribution or friction coefficients, enabling the prediction of manipulation action effects and the planning of safe bimanual manipulation strategies. Temtsin and Degani, (2017) compared multiple decision-making algorithms for autonomous pile disassembling, including approaches based on potential energy, object encapsulation, and explicit support relationships, and demonstrated robust performance under both full and partial knowledge conditions. While these geometric and rule-based methods provide valuable frameworks for analyzing structural dependencies, they typically rely on explicitly defined support rules and geometric constraints. In contrast, physics-based simulation can directly evaluate structural stability through dynamic modeling, offering a complementary approach that naturally captures complex multi-body interactions without requiring hand-crafted support relation rules.

Our work bridges these approaches by reconstructing an approximate 3D scene using a real-to-sim pipeline and employing real-time simulation to evaluate extraction strategies. This unified pipeline avoids reliance on pre-trained object models or synthetic datasets (Motoda et al., 2022; Motoda et al., 2023) and generalizes across both structured and unstructured warehouse environments.

## 3 Methodology

This section presents a physics-aware grasp planner—an end-to-end pipeline that processes visual inputs to generate an executable grasp pose sequence. Additionally, two stacked object retrieval approaches are presented: physics-aware and heuristic-based. These approaches are applied to two key tasks: (1) single-box extraction and (2) shelf clearance. The physics-aware approach utilizes simulation to predict potential collapses and collisions, while the heuristic-based approach serves as a baseline for performance evaluation. The following sections provide a detailed breakdown of each component within the proposed planner.

### 3.1 Real-to-sim pipeline

**FIGURE 3:**
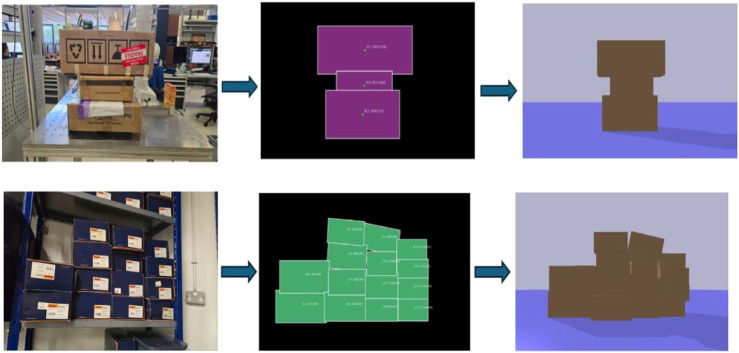
The real-to-sim pipeline converts single modeling images into approximate 3D simulation environments for physics-based planning.

First, a single modeling image is captured using a vision sensor, followed by object segmentation. From the resulting mask, features like minimum oriented bounding boxes and centroids are computed, providing estimates of each box’s dimensions, location, and orientation. Finally, integrating the segmentation mask with the depth map, the distance of each box from the camera is also determined. This enables the reconstruction of the observed scene to a 3D simulation space as seen in Figure 3. This forms the basis of the physics-aware approach. By simulating the removal of a specific box, the system can predict its impact on the overall stability of the stack. Details of the real-world implementation are provided in Section 4.

The output of the perception pipeline reconstructs the approximate positions and orientations of the detected cardboard boxes within the simulation environment. Because the approach relies on a single modeling image, it is not possible to accurately estimate the depth dimension of each box. However, precise depth measurements are not critical for this approach, which is to capture the relative spatial relationships between boxes in the stack rather than to model them with exact dimensions. To accommodate this uncertainty, the depth of each box is randomized, with the maximum depth constrained by the depth of the shelf. Moreover, the simulation is run ten times, each iteration randomizing the depth values to reflect the natural variability encountered in real-world conditions. This method ensures that the simulation robustly represents the interactions among boxes, enabling reliable predictions of how the removal of any given box will affect the overall stability of the stack.

**FIGURE 4:**
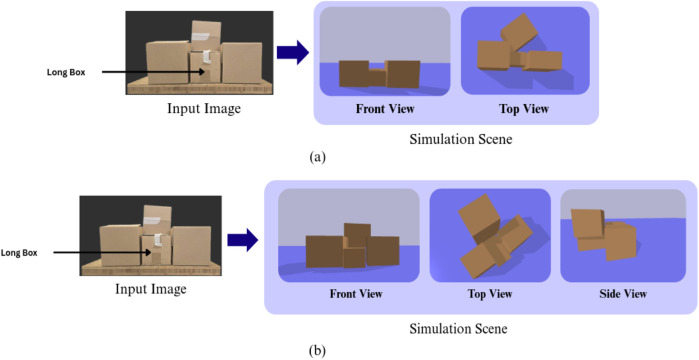
**(a)** Without Estimation: Single-view noise leads to an underestimated depth (blue box), causing the top box (red) to spawn in mid-air and resulting in an unrealistic simulation collapse. **(b)** With Estimation: Leveraging inventory priors corrects the supporting box’s depth, ensuring a stable, physically-accurate scene initialization for safe collapse prediction.

For deployment in real-world environments such as warehouses, we propose a robust box-depth estimation strategy that leverages known inventory data, taking advantage of the fact that the available box types and quantities are typically predefined. By estimating only the visible length and width of a detected box from single-view depth camera measurements and using the inventory dataset as a prior, the system predicts the most probable missing depth dimension. This reduces ambiguity caused by occlusion and improves the reliability of downstream physics simulation and extraction-strategy generation. As demonstrated in Figure 4, omitting this estimation leads to incorrect scene initialization: an underestimated depth for a supporting box leaves the top box with no surface to rest on, causing an immediate and unrealistic collapse at the start of the simulation. When the proposed estimation is applied, the correct depth of the supporting box is inferred, allowing the top box to rest as it does in the real-world and ensuring that the simulated scene is accurate, stable, and suitable for generating valid extraction strategies.

### 3.2 Simulation properties and considerations

PyBullet is used as the physics engine due to its efficiency and ease of integration. Gravity is set to 9.81 m/s^2^, with box density defined as 1 kg/m^3^ and mass assumed to be uniformly distributed for consistent collision responses. The surface friction coefficient is set to 0.75, while spinning friction is 0.01, to ensure realistic interactions. Friction along the surface is assumed to be constant across all boxes to standardize contact behavior. These parameters enable a more realistic simulation of stacked cardboard boxes.

**FIGURE 5:**
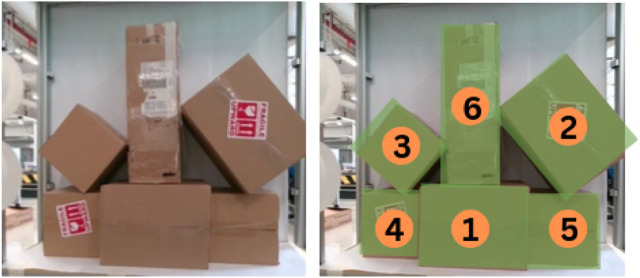
The image on the left shows the real-world scenario of stacked boxes, and the image on the right shows the corresponding segmentation mask with labeled Box IDs.

It is important to note that the primary role of the physics engine here is to model inter-dependencies (i.e., identifying which boxes support others) rather than to model precise collapse dynamics or trajectories. Sensitivity analyses conducted during development (as seen in Table 1; Figure 5) revealed that while varying mass and friction parameters changed the speed of a collapse, the binary state of stability (stable vs. unstable) remained consistent. Therefore, standardizing these parameters does not adversely affect the planner’s ability to generate safe extraction sequences.

**TABLE 1:** Robustness of the Physics-Aware Extraction Strategy.

Run no.	Action plan generated
1	2, 3, 6, 4, 1, 5
2	3, 2, 6, 5, 1, 4
3	2, 3, 6, 1, 5, 4
4	3, 2, 6, 1, 5, 4
5	3, 2, 6, 1, 4, 5
6	2, 3, 6, 4, 1, 5
7	3, 2, 6, 4, 1, 5
8	3, 2, 6, 1, 5, 4
9	2, 3, 6, 4, 1, 5
10	2, 3, 6, 4, 1, 5

The table shows the action plans generated across 10 simulation trials with randomized physical parameters (mass, friction, and size). The results confirm the algorithm’s robustness: in all 10 trials, the critical structural dependencies (Box 2; Box 3 before Box 6; Box 3 before Box 4; Box 2 before Box 5) remained invariant. This validates that the approach successfully determines the safe extraction topology without requiring precise physical measurements.

### 3.3 Collapse detection and simulating box removal

**FIGURE 6:**
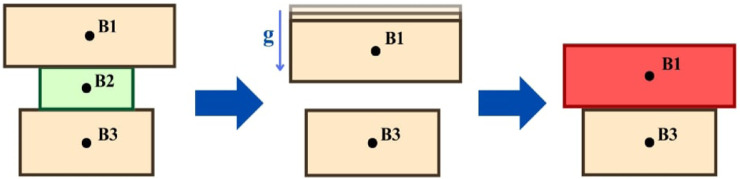
Collapse detection during box removal, highlighting the impact when Box B2 is removed.

A box is considered to have collapsed if the simulation detects an unexpected increase in linear or angular velocity beyond a predefined threshold. This enables the simulation to precisely determine the consequences of removing a box in real time. This can be seen in Figure 6.

To further enhance realism, random disturbances and vibrations are introduced during the removal process, accounting for unintended forces that may affect neighboring boxes. Additionally, boxes are removed from the stack by exerting a forward pull on them from their centroids. This is done to mimic how a suction gripper would pull these boxes out. This enables more accurate modeling of how drag and friction affect the stack during the extraction process.

### 3.4 Single-box removal using the physics-aware approach

In this task, the goal is to extract a defined target box without causing any unintended collapses or collisions. For this, the physics-aware approach utilizes a backtracking algorithm to determine the optimal sequence for removing stacked boxes without causing unintended collapses. The process begins with a user-specified target box, which is the box that needs to be safely extracted. The following explains each step of the algorithm in more detail:

1. Initial Removal Attempt: The simulation attempts to remove the target box. If this removal does not cause any structural collapse (i.e., no box shifts beyond a predefined movement threshold), the process is complete. However, if a collapse occurs, the simulation resets, and a list of affected (collapsed) boxes is generated.
2. Backtracking to Find a Safe Path: From the list of collapsed boxes, a random box (typically the first collapsed box) is selected and removed first. The simulation then checks for any additional collapses. If another collapse occurs, the process repeats: a new list of affected boxes is generated, and another box is removed. This iteration continues until a box is removed that does not cause further collapses.
3. Reattempting Target Box Removal: Once a stable state is reached (where removing a box does not trigger further collapses), the algorithm retries removing the target box. If removing the target box still leads to a collapse, the backtracking process continues, adjusting the removal order. This recursive approach ensures that boxes are removed in a sequence that prevents structural instability.
4. Final Removal Order: The algorithm continues this process until the target box can be safely removed without triggering collapses. The final sequence of removals represents the optimal order for real-world execution. This algorithm is illustrated in Figure 5.

Algorithm 1 Single-Box Removal Using the Dynamic Simulation.

**Input:** modeling image and target box
  
ActionPlan← Start with an empty array
  
action← target box
  **while** Solution is not found **do**
    removeBox(action) ← Remove Box as defined by action variable
    **if** collapse is detected **then**
      Remove action from ActionPlan
      
action← First box that collapses
      
resetSimulation()

    **end if**
    **if** (collapse is not detected) and (action ≠ target box) **then**
      Add action to ActionPlan
      
action← target box ← Try to remove target box again
    **end if**
    **if** (collapse is not detected) and (action = target box) **then**
      Solution is Found!
    **end if**
  **end while**
  **return** ActionPlan

### 3.5 Shelf clearance using the dynamic simulation

The goal of this task is to remove all the boxes in a stack without causing collapse or collisions. Here, the proposed approach evaluates each box and removes it one at a time, followed by a stability check to detect any unintended changes in the stack. If removal causes instability, such as tilting or shifting, the box is skipped, and the algorithm moves to the next one. If removing a box results in a complete stack collapse, the simulation resets to its previous state, and that box is permanently skipped.

**FIGURE 7:**
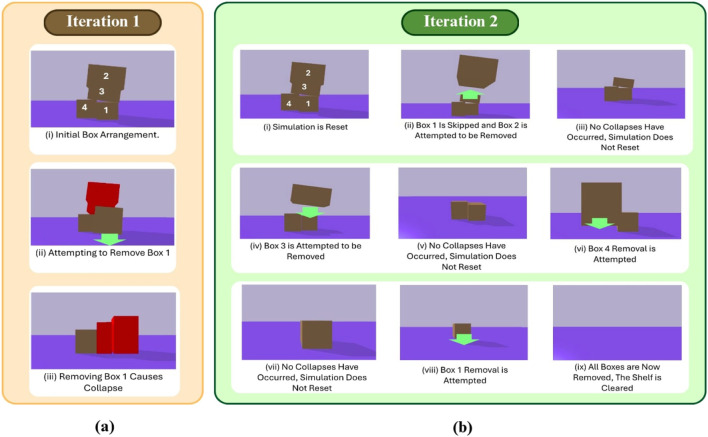
Illustration showing first **(a)** and second **(b)** iterations of the shelf clearing algorithm.

**FIGURE 8:**
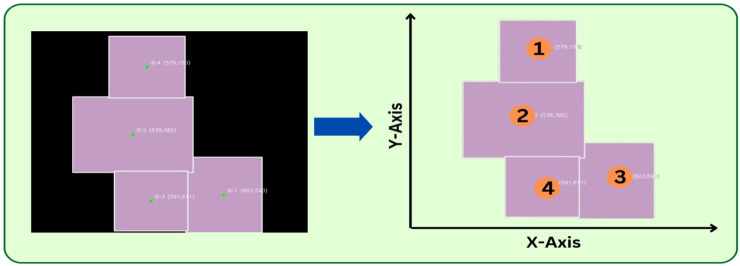
Illustration showing the action plan generated by the base-heuristics approach for a shelf-clearing task.

If the removal is stable, the box is successfully extracted, and the algorithm continues. This process repeats until all boxes have been evaluated, with only the stable ones being extracted. The algorithm then revisits any skipped boxes and re-evaluates them, following the same checks, until all boxes are removed. Figures 7, 8 illustrate an overview of the algorithm.

Algorithm 2 Shelf clearance using the dynamic simulation.

**Input:** modeling image
  
DetectedBoxes← List of tags of the boxes as detected by the vision pipeline
  
ActionPlan← Start with an empty array
  
action← first item in DetectedBoxes list
  
SkippedBoxes← Start with an empty array
  **while** Solution is not found **do**
    **if** action is not in SkippedBoxes **then**
      removeBox(action) ← Remove Box as defined by action variable
    **end if**
    **if** collapse is detected **then**
      Remove action from ActionPlan
      Add action to SkippedBoxes list
      
action← Next box in the DetectedBoxes list
      
resetSimulation() ← Resets Simulation
    **end if**
    **if** (collapse is not detected) and (action ≠ Last Box in DetectedBoxes List) **then**
      Add action to ActionPlan
      
action← Next box in the DetectedBoxes list
    **end if**
    **if** (collapse is not detected) and (action = Last Box in DetectedBoxes List) **then**
      **if** (SkippedBoxes list is not Empty) **then**
        
SkippedBoxes← Empty Array
        
resetSimulation()

      **end if**
      **if** SkippedBoxes list is Empty **then**
        Solution is Found! ← Stop the while loop
      **end if**
    **end if**
  **end while**
  **return** ActionPlan

### 3.6 Heuristic-based sequence planning

**FIGURE 9:**
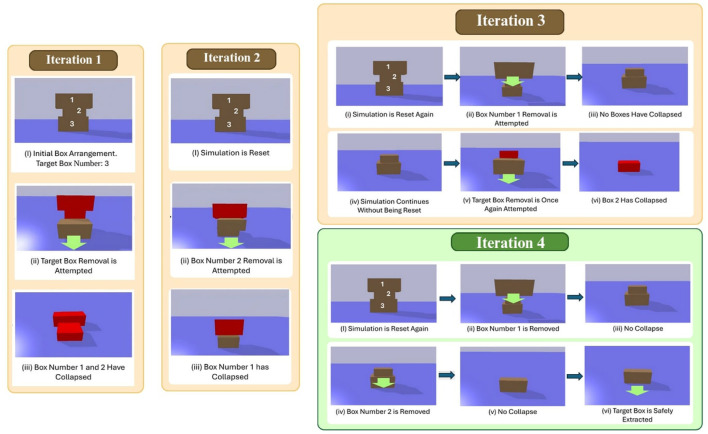
Overview of the single-box extraction algorithm.

This method employs a simple heuristic that considers only the vertical positioning of boxes relative to the ground. It sorts all detected boxes based on their y-axis coordinates (as seen in Figure 9), prioritizing those positioned highest in the stack. The system then sequentially removes boxes until it reaches the target. While more sophisticated geometric reasoning methods exist for analyzing support relations (Mojtahedzadeh et al., 2015; Kartmann et al., 2018; Temtsin and Degani, 2017), this height-based heuristic represents a practical baseline commonly employed in simple warehouse picking implementations, where boxes are removed from top-to-bottom based on accessibility. This approach is specifically designed to serve as a baseline for comparing the performance of the proposed physics-aware approach and evaluating its effectiveness in more complex scenarios. For shelf clearance, the algorithm continues to remove boxes until the shelf is empty. This can be seen in Figure 8.

While we acknowledge the existence of advanced grasping planners in the literature (e.g., Motoda et al., 2021; Bejjani et al., 2021), direct experimental comparison is infeasible due to fundamental hardware and systemic differences. First, many state-of-the-art methods utilize bi-manual manipulation or multi-finger grippers, whereas our system employs a single-arm setup with a suction gripper. Second, learning-based approaches often rely on pre-trained object models or extensive synthetic datasets, which contradict our approach’s design for zero-shot operation without object-specific priors. Finally, alternative geometric methods often require multi-view perception systems, while our framework is optimized for single-view depth sensing. Consequently, the height-based heuristic is selected as the most representative baseline for standard industrial single-arm suction picking.

Algorithm 3 Single-box Extraction Using Heuristics.

 **Input:** RGB Image and Target Box
 
positions← Coordinates of the centroids of all detected boxes
 
SortedPositions← sorted positions ← Sort positions from highest to lowest
 
ActionPlan← Start with an empty array
 **for** **each** item in SortedPositions **do**
   **if** action≠target **then**
      Add action to ActionPlan

   **end if**
   **if** action=target then
   **end if**
 **end for**
 **return** ActionPlan

## 4 Experiments and results

### 4.1 Experimental setup

**FIGURE 10:**
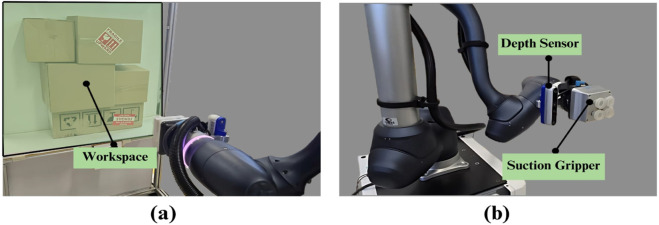
Experimental setup. **(a)** Side view. **(b)** Front view.

The experimental setup (shown in Figure 10) features a Doosan H2515 6-degree-of-freedom (6DOF) robotic arm, equipped with a suction gripper for precise box handling, and an Intel RealSense D455 depth camera for real-time spatial perception. A 0.1 m × 0.3 m × 0.16 m shelf, positioned 0.104 m from the robot, served as the designated area for stacking boxes and was used for both single-box extraction and shelf clearance experiments. To demonstrate the robustness of the proposed approach, mixed-sized cardboard boxes were utilized to create the stacking scenes. Specifically, three types of cardboard boxes with varying dimensions—0.23 m × 0.31 m × 0.25 m, 0.20 m × 0.20 m × 0.20 m, and 0.50 m × 0.17 m × 0.17 m—were selected. These different box sizes introduce variability in the stacking environment.

Each experimental configuration (structured and unstructured, for both single-box extraction and shelf clearance) was conducted with consistent stacking arrangements to ensure reproducibility. The real-world experimental results presented represent stable outcomes verified through multiple observation runs to confirm the reliability of the physics simulation predictions.

### 4.2 Considered stacking complexities

**FIGURE 11:**
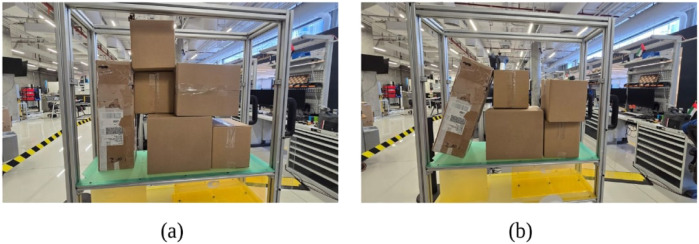
Different stacking complexities considered. **(a)** Structured cluttered stack. **(b)** Unstructured cluttered stack.

To better define the problem space, this study categorizes stacked structures into two distinct types:

1. Structured Cluttered Stacks–While these stacks maintain a general order, they introduce variations in depth, meaning some boxes extend further out than others. This uneven layering creates partial obstructions, reducing visibility and making removal more challenging. This is shown in Figure 11a.
2. Unstructured Cluttered Stacks - These are more complex, consisting of randomly placed boxes with varying orientations, rotations, and significant depth variations. The unpredictability of these stacks increases the risk of collapse when boxes are removed. This is shown in Figure 11b.

### 4.3 Training settings

As explained in the previous section, the boxes are first perceived by the camera and then placed into the simulation space. To achieve this, a single modeling image is captured using an Intel RealSense D455 depth camera. Object segmentation is performed using a custom-trained YOLOv11n-seg model Jocher and Qiu (2024) trained on the Online Stacked Cardboard Boxes Yang et al. (2021), comprising 8,401 images. The dataset was split into a training set (80%), validation set (10%), and test set (10%) to ensure robust model evaluation. Given the dataset’s limited variety in box orientations, data augmentation was employed to improve the model’s ability to handle angled boxes. Specifically, random rotations ranging from −90 to 90° were applied to the training images to simulate different box angles and enhance generalization. The model was trained on a system equipped with an RTX 3070 Ti (8GB VRAM), paired with an Intel i7-11700K processor and 64GB of RAM. During training, the model achieved a Mean Average Precision (mAP) of 0.87 on the test set, with validation performance consistently matching the test set results, indicating the model’s generalization capability.

### 4.4 Results

**FIGURE 12:**
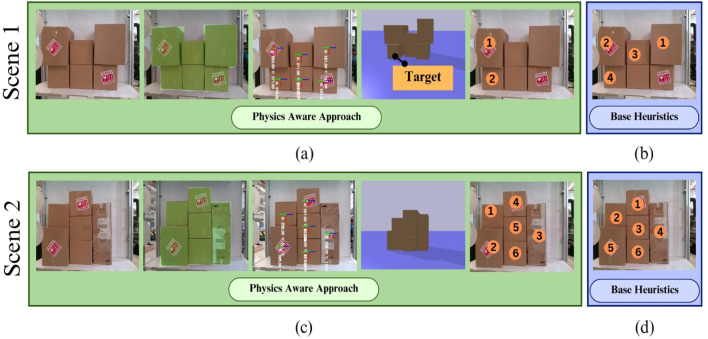
Sequence planning for structured stack experiments: **(a)** illustrates the physics-based approach for the extraction task, **(b)** presents action sequences predicted by the base-heuristics approach, **(c)** depicts the physics-aware method’s action sequence for shelf clearance, and **(d)** shows the output predicted by the base-heuristics approach.

**FIGURE 13:**
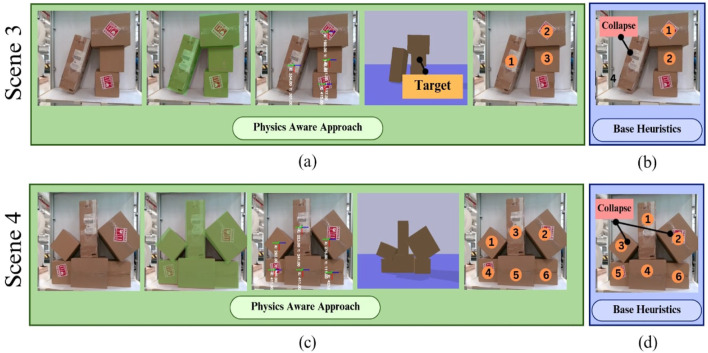
Sequence planning for unstructured stack experiments: **(a)** demonstrates the physics-based approach for the extraction task, **(b)** showcases action sequences predicted by the base-heuristics approach, **(c)** illustrates the physics-aware method’s action sequence for shelf clearance, and **(d)** presents the output predicted by the base-heuristics approach.

Experiments were conducted testing the efficacy of the proposed approach on two tasks: (1) single-box extraction and (2) shelf clearance. In the single-box extraction task, the objective was to remove a specific target box without causing any collapse or collision. In the shelf clearance task, the goal was to efficiently extract all boxes from a shelf while maintaining structural integrity. The study conducted eight experiments across two tasks and two stacking conditions using both physics-aware and base-heuristics approaches as follows:

1. Scene 1: single-box extraction in structured stacks using a physics-aware and base-heuristics approach, as seen in Figures 12a,b.
2. Scene 2: Shelf clearance in structured stacks using a physics-aware and base-heuristics approach, as seen in Figures 12c,d.
3. Scene 3: single-box extraction in unstructured stacks using physics-aware and base-heuristics approaches as seen in Figures 13a,b.
4. Scene 4: Shelf clearance in unstructured stacks using physics-aware and base-heuristics approaches as seen in Figures 13c,d.

The results demonstrate that in structured stacks, both methods extract the target box successfully, although the physics-aware approach achieves higher efficiency by removing fewer extraneous boxes. In structured shelf clearance, both methods clear the shelf without incident. However, in unstructured stacks, the base-heuristics approach frequently fails to extract the target box and clear the shelf, whereas the physics-aware approach consistently achieves successful outcomes. This is because the physics-aware approach allows for a more accurate modeling of the relationships between boxes in the stack. As a result, the algorithm can detect potential collapses even when the affected boxes are not directly stacked on top of one another. The results from these experiments are shown in Tables 2, 3.

**TABLE 2:** Experimental results for single-box extraction tasks.

Condition	Approach	Result	Time (s)	Boxes removed
Structured	Physics-aware	✓	43	2
	Base-heuristics	✓	88	4
Unstructured	Physics-aware	✓	75	3
	Base-heuristics	Fail	38	2

**TABLE 3:** Experimental results for shelf clearance tasks.

Condition	Approach	Result	Time (s)
Structured	Physics-aware	✓	116
	Base-heuristics	✓	98
Unstructured	Physics-aware	✓	110
	Base-heuristics	Fail	25

### 4.5 Scalability

**FIGURE 14:**
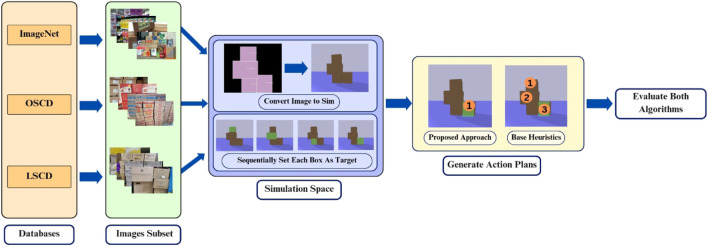
Overview of the scalability experiments.

Following the real-world experimental results, evaluation on three diverse datasets was conducted to assess the scalability and performance of the proposed approach relative to base-heuristics. The evaluation used 500 images from ImageNet (Deng et al., 2009), 1,000 images from the Online Stacked Carton Dataset (OSCD) (Yang et al., 2021) (which consisted solely of images from the test and validation sets) and 1,000 images from the Live Stacked Carton Dataset (LSCD) (Yang et al., 2021). For each image, the vision pipeline converted the scene into a simulation environment. In each simulation, every box was sequentially designated as the target. For each target, both the physics-aware approach and base-heuristics generated action plans, which were executed in the simulation to evaluate efficiency and detect any unintended collisions or collapses. Efficiency was calculated using Equation 1. The results are collated in Table 4, with an overview shown in Figure 14.

$$\text{Efficiency Improvement }\left(\%\right)=\left(\frac{T_{bh}-T_{pa}}{T_{pa}}\right)\times 100$$ (1)

**TABLE 4:** Comparison of base-heuristics and Physics-Aware Approach with absolute and relative metrics.

Data subset	Base-heuristics (B.H)		Physics-aware approach			
	Boxes removed	Time (s)	Boxes removed	Time (s)	Success rate (%)	Efficiency gain (%)
ImageNet Deng et al. (2009) (500 images)	4.98	56.16	2.51	37.56	97 (vs. 68 B.H)	+49.52
OSCD Yang et al. (2021) (1,000 images)	5.09	64.08	2.57	32.06	92 (vs. 60 B.H)	+96.76
LSCD Yang et al. (2021) (1,000 images)	4.94	62.62	2.55	31.70	89 (vs. 55 B.H)	+94.56

Here, $T_{bh}$ is the average time taken by the base-heuristics approach and $T_{pa}$ is the average time taken by the physics-aware approach.

For the dataset evaluation, ground truth labels regarding collapse outcomes and safe extraction orders were manually annotated by human reviewers to ensure an accurate comparison against the simulation and baseline outputs.

### 4.6 Discussions

The physics-aware approach consistently outperformed base-heuristics across environments and datasets. In structured settings, both methods completed tasks. Still, the physics-aware approach was significantly more efficient, extracting boxes in 43 s with minimal disruption (removing only two boxes) versus 88 s and four removals for base-heuristics. This efficiency gap widened in unstructured environments, where base-heuristics failed, while the physics-aware approach remained reliable.

Scaling across datasets reinforced these trends. As shown in Table 4, the physics-aware method consistently removed fewer boxes, completed tasks nearly twice as fast, improved success rates by up to 61.2%, and achieved a retrieval efficiency of up to 96.76%.

While base-heuristics perform in structured environments, they struggle with real-world complexity. By modeling indirect interactions, the physics-aware approach proves more robust and scalable for robotic manipulation. Minor segmentation inaccuracies from single-depth perception can introduce occasional errors, but overall, integrating physical modeling significantly enhances efficiency and reliability. The robust box-depth estimation strategy using inventory priors substantially mitigates these perception errors by ensuring that supporting boxes are reconstructed with physically plausible dimensions in the simulation environment.

## 5 Conclusion

This paper presented a physics-aware grasp planner for retrieving diversely cluttered stacked boxes from shelves. Real-world experiments using a robotic manipulator equipped with a suction gripper compared and validated the practical effectiveness of the proposed physics-aware and heuristics-based approaches for safe shelf picking. Although the current implementation uses a suction gripper, the proposed physics-aware approach can also be extended to other grasping pipelines and end-effectors. The experiments show that in structured stacks, the base-heuristics approach is less efficient, and in unstructured conditions, it fails completely. While the physics-aware approach proved more efficient and successful, it required longer computation times than the base-heuristics method. Additionally, our current implementation relies on single-view depth sensing, which introduces limitations in handling severe occlusions where objects completely obstruct others from view.

Additionally, evaluations on large-scale existing datasets showed that the physics-aware approach consistently outperforms the base-heuristics approach, achieving up to 61.2% higher success rates and 96.76% greater efficiency, particularly in unstructured settings.

Future work will integrate multi-modal perception (vision, tactile, force) and active re-planning to support in-contact tasks. To address the occlusion limitations inherent in single-view depth sensing, we plan to incorporate multi-view perception or active camera repositioning strategies. We also plan to develop human–robot collaborative modes, where the robot adapts its grasp strategy in response to human gestures or semi-autonomous shared control in smart factory cells.

Future work might also address the variation in weight and density of the boxes and evaluate how the proposed approach could be enhanced to deal with such realistic scenarios.

## Data Availability

The original contributions presented in the study are included in the article/supplementary material, further inquiries can be directed to the corresponding author.
